# Rhamnolipids Produced by Indigenous *Acinetobacter junii* from Petroleum Reservoir and its Potential in Enhanced Oil Recovery

**DOI:** 10.3389/fmicb.2016.01710

**Published:** 2016-11-07

**Authors:** Hao Dong, Wenjie Xia, Honghong Dong, Yuehui She, Panfeng Zhu, Kang Liang, Zhongzhi Zhang, Chuanfu Liang, Zhaozheng Song, Shanshan Sun, Guangqing Zhang

**Affiliations:** ^1^State Key Laboratory of Heavy Oil Processing, College of Chemical Engineering, China University of PetroleumBeijing, China; ^2^Power Environmental Energy Research Institute, CovinaCA, USA; ^3^College of Chemistry and Environmental Engineering, Yangtze UniversityJingzhou, China; ^4^Dalian Design Branch, China Petroleum Engineering and Construction CorporationDalian, China; ^5^State Key Laboratory of Heavy Oil Processing, Faculty of Sciences, China University of PetroleumBeijing, China; ^6^School of Mechanical, Materials and Mechatronic Engineering, University of Wollongong, WollongongNSW, Australia

**Keywords:** biosurfactant, *Acinetobacter junii*, ESI FT-ICR-MS, biodegradation, polar components, visualization micromodel, microbial enhanced oil recovery

## Abstract

Biosurfactant producers are crucial for incremental oil production in microbial enhanced oil recovery (MEOR) processes. The isolation of biosurfactant-producing bacteria from oil reservoirs is important because they are considered suitable for the extreme conditions of the reservoir. In this work, a novel biosurfactant-producing strain *Acinetobacter junii* BD was isolated from a reservoir to reduce surface tension and emulsify crude oil. The biosurfactants produced by the strain were purified and then identified via electrospray ionization-Fourier transform ion cyclotron resonance mass spectrometry (ESI FT-ICR-MS). The biosurfactants generated by the strain were concluded to be rhamnolipids, the dominant rhamnolipids were C_26_H_48_O_9_, C_28_H_52_O_9_, and C_32_H_58_O_13_. The optimal carbon source and nitrogen source for biomass and biosurfactant production were NaNO_3_ and soybean oil. The results showed that the content of acid components increased with the progress of crude oil biodegradation. A glass micromodel test demonstrated that the strain significantly increased oil recovery through interfacial tension reduction, wettability alteration and the mobility of microorganisms. In summary, the findings of this study indicate that the newly developed BD strain and its metabolites have great potential in MEOR.

## Introduction

Water flooding is an efficient oil recovery technology employed worldwide. However, a large amount of petroleum still remains underground. To enhance oil recovery, various approaches have been developed to improve the oil recovery, such as chemical flooding, CO_2_ foam flooding, and steam flooding. However, these technologies require substantial effort and have higher energy, economic and environmental costs. Therefore, microbial enhanced oil recovery (MEOR), an economically efficient and environmentally friendly technology, has gained increasing attention in academic and industrial fields. MEOR technology is an economical and environmentally friendly tertiary recovery method that utilizes microorganisms and their metabolites in oil reservoirs to achieve increased oil recovery efficiency and longer exploitation of oil reservoirs (Sun et al., 2011; Xia et al., 2011; Gudiña et al., 2013). The suggested mechanisms of MEOR include: reduction of interfacial tension (IFT), wettability alteration, changes in flow pattern, gas production, oil emulsification, and oil viscosity reduction (Karimi et al., 2012; Nazina et al., 2013; Rabiei et al., 2013; Xiao et al., 2013; Li et al., 2014; Sarafzadeh et al., 2014). Among these mechanisms, two are thought to be the main mechanisms behind MEOR success. The first mechanism involves the reduction of the IFT induced by biosurfactants. Low liquid–liquid IFT is important in promoting emulsification and improving the mobility of crude oil. The second mechanism is wettability alteration (Afrapoli and Alipour, 2012; Rabiei et al., 2013; Chandankere et al., 2014). In general, biosurfactant producers are considered a key factor in enhancing oil recovery.

In the past decades, a number of studies have reported the capability of bacteria to utilize different carbon sources to produce biosurfactants and their application in the petroleum industry (Pereira et al., 2013; Nalini and Parthasarathi, 2014; Domingos et al., 2015; Marti et al., 2015). The biosurfactant producers are mainly categorized into two classes, low-molecular-weight biosurfactant producers and high-molecular-weight bioemulsifier producers (Rosenberg and Ron, 1999). Low-molecular-weight biosurfactant producers are mainly *Pseudomonas. Bacillus. Rhodococcus*, and *Nocardia* species, which can produce different kinds of glycolipids and lipopeptides (Cooper and Goldenberg, 1987; Xia et al., 2014; Liu et al., 2015). High-molecular-weight bioemulsifier producers are mainly *Acinetobacter. Bacillus*, and *Geobacillus* species, which can produce lipopolysaccharides and glycoproteins (Zheng et al., 2011; Yadav et al., 2012). These metabolic products play indispensable roles in multiple mechanisms for improving oil recovery (Dastgheib et al., 2008; Zheng et al., 2011). Indigenous microbes, which are better adapted to the oil reservoir environment, have been used in MEOR processes (Castorena-Cortés et al., 2012). Numerous indigenous species with the capability to degrade crude oil efficiently and produce low-molecular-weight biosurfactants, such as *Pseudomonas. Bacillus. Rhodococcus*, and *Arthrobacter* species, play a dominant role in enhancing oil recovery. Nevertheless, studies on *Acinetobacter* species isolated from reservoirs and its feasibility of MEOR are scarce, although it is a dominant species in many reservoirs (Zhang et al., 2010; Lenchi et al., 2013; You et al., 2016). Rhamnolipids are well-known glycolipid surfactants produced by *Pseudomonas* with a great quantity, exhibiting excellent performance in reducing surface (interfacial) tension and changing emulsification and wettability (Orathai et al., 2007). Successful applications of *Pseudomonas* species in the petroleum industry and environmental remediation have been widely documented (Pasumarthi et al., 2013; Xia et al., 2014; Zuo et al., 2015). However, some *Pseudomonas* species are opportunistic human or plant pathogens. Therefore, non-pathogenic and rhamnolipid-producing bacterial strains are still the targets of novel investigations in the academic and industrial community of petroleum.

Low-molecular-weight biosurfactants are usually generated during the utilization of hydrophobic feedstocks; therefore, the occurrence of crude oil biodegradation by indigenous microorganisms is generally detected during the MEOR process. The alteration of saturated and aromatic hydrocarbons during biodegradation has been reported (Zheng et al., 2011; Gudiña et al., 2013; Xia et al., 2014). However, studies on the alteration of non-hydrocarbons during MEOR processes have not been reported. According to Li et al. (2010) and Kilpatrick (2012), non-hydrocarbon compounds comprising a large percentage of crude oil are the main interfacial active components. Oil emulsification is closely related to non-hydrocarbon compounds (Pan et al., 2013). Oxygenated compounds are the most important polar group in the oil component and are closely related (Pan et al., 2013); additionally, oxygenated polar compounds have an important role in oil emulsification and wettability alteration in oil reservoirs (Kilpatrick, 2012). Nevertheless, the alteration of these compounds and its effects on the oil recovery during MEOR have not been extensively explored.

In the present study, a non-pathogenic, hydrocarbon-degrading bacterial strain, *Acinetobacter junii* BD, was isolated which has been demonstrated to produce biosurfactants with good potential for MEOR. This strain was able to emulsify oil in water by reducing the IFT between oil and water. The structural diversity of the produced biosurfactants was analyzed, and the strain was able to degrade crude oil and produce rhamnolipid surfactants. The biosurfactants and biomass yields were optimized. The recovery of residual oil was examined with a glass micromodel.

## Materials and Methods

### Isolation and Identification of Microorganisms

The oil and formation water samples used in this study were collected from oil wells at Xinjiang Oilfield, which is located in Northwest China (45.41336°N, 85.04578°E). The depth of the petroleum reservoir was 1088 m, with a temperature of 32°C. Oil and formation water were collected at the wellheads after connection lines were flushed for 5 min prior to filling a 15 L sterilized plastic container. A 5-mL sample was then cultivated in 100 mL mineral enrichment medium (MEM) amended with 10 mL crude oil (from the same oil well). This was carried on under aerobic condition at 37°C for 7 days with agitation at 150 rpm. 100 μL of culture supernatant was then collected, spread on Luria Broth (LB) agar plates and grown for 3 days. The microbial colonies obtained were further purified by streaking on LB agar and cultivating the strains with ability of hydrocarbon emulsification and surface tension reduction. The emulsification activity of the biosurfactants was evaluated by emulsification index (E_24_), which was quantified as the ratio of the emulsified volume to the total volume of a mixture containing 5 mL bioemulsifier solution and 5 mL of n-hexadecane after it was vortexed for 5 min and then settled for 24 h (Zheng et al., 2011). The surface tension was determined using a surface tensiometer JK99B (POWEREACH, China). The strains BD was selected because of its superior performance in hydrocarbon emulsification and reduce surface tension, and its growth was assessed at wide ranges of temperatures, pH values and salinities. The MEM contained 6 g/L NaNO_3_, 1 g/L KH_2_PO_4_, 1 g/L K_2_HPO_4_, 0.5 g/L MgSO_4_, 0.02 g/L FeSO_4_, and 0.02 g/L Na_2_MoO_4_ at a pH of 7.0–7.2.

The morphological, physiological, and phylogenetic characteristics of the isolated strains were analyzed following Bergey’s Manual of Systemic Bacteriology and SSU rRNA sequencing, as previously described by Chen et al. (2012).

### Effects of Carbon and Nitrogen Sources on Biosurfactant Production

The growth of strain BD and biosurfactant production were evaluated using a mineral salt medium (MSM) with different carbon and nitrogen sources. The MSM contained 1.0 g/L K_2_HPO_4_, 1.0 g/L KH_2_PO_4_, 0.25 g/L MgSO_4_, 0.02 g/L Na_2_Mo_2_O_4_, and 0.02 g/L FeSO_4_. Glucose, hexadecane, molasses, soybean oil, glycerol, paraffin, sucrose, ethanol, and diesel oil were evaluated as carbon sources while 8 g/L NaNO_3_ was used as a nitrogen source. These carbon sources were added into the MSM at a concentration of 10 g/L. The following compounds were tested as nitrogen sources while soybean oil (10 g/L) was used as a carbon source: NaNO_3_, NH_4_NO_3_, (NH_4_)_2_SO_4_, NH_4_Cl, beef extract, tryptone, and urea.

The optimization experiments were carried out in 250-mL flasks containing 100 mL of medium. Each flask was inoculated with 1% of a pre-culture grown in the same medium for 24 h. The flasks were incubated at 37°C for 120 h with agitation at 150 rpm. The cells were then harvested by centrifugation (at 10,000 rpm for 20 min), and the cell weight after drying at 105°C for 48 h was determined (Pereira et al., 2013). The cell-free supernatants were used to measure the biosurfactant yield. The biosurfactant yield was derived from a standard curve prepared with rhamnose and expressed as rhamnose equivalents (Vyas and Dave, 2011). All optimization experiments were conducted in triplicate, and the data are presented as the means.

### Biosurfactant Extraction

The cell-free supernatant was obtained by centrifugation at 10,000 rpm for 20 min at 4°C. After the pH was adjusted to 2.0 with 1 mol/L HCl, 200 mL of the samples were extracted twice with equal volume of ethyl ether. The solvent was removed by vacuum distillation, to collect the dry product. The purified biosurfactants in chloroform were then spotted on a silica gel thin-layer chromatography (TLC) plate (Silica gel 60, Merck, Qingdao, China). The compounds were separated using a mobile phase containing chloroform, methanol, acetic acid, and water in volume ratios of 65:25:1:1, all of the solvent components being of analytical grade purity. The dry plates were sprayed with a phenol sulfate solution and incubated at 105°C for 5 min for glycolipid detection.

### Purification and Structural Analysis of the Biosurfactants

The crude extracts of biosurfactants were dissolved in chloroform and were then loaded onto a column of silica gel (100–200 meshes; all silica separation materials used were from Qingdao Marine Chemical, Co. Ltd, Qingdao, China). The column was first washed with n-hexane and then with chloroform/methanol solvent systems to recover potential biosurfactants. The eluate was collected separately every 10 mL. The glycolipid was detected using phenol-sulfate colorimetry. The chemical composition of each component fractionated from the purified glycolipid was then preliminarily investigated via electrospray ionization (ESI) Fourier transform ion cyclotron resonance mass spectrometry (ESI FT-ICR-MS). Each mass typically yielded a unique molecular formula within a mass tolerance of 1.0 ppm with a ^13^C-isotope because of high mass accuracy. Nevertheless, the molecular formulas must be validated using the rules described by Lin et al. (2014). The sum formulas of the true discriminant masses were calculated and validated through isotope pattern matching using Bruker Daltonics Data Analysis version 3.4. The molecular formulas were then validated using the ChemSpider database.

### Biodegradation of Crude Oil by Strain BD

Bacterial cells from a 5-mL LB culture were harvested by centrifugation at 10,000 rpm for 2 min, washed in triplicate with sterilized saline and inoculated into 100 mL MEM (pH: 6.8–7.2) supplemented with crude oil in a concentration of 0.5% (w/v). One culture containing crude oil but without cells was used as control. The flasks were then separately incubated in a rotary shaker at 150 rpm for 2 or 14. The oil from the liquid culture was extracted three times with dichloromethane.

### Analysis of Polar Components during Crude Oil Biodegradation

The polar components of the crude oil were examined using ESI FT-ICR-MS (Shi et al., 2010a). The extracted crude oil was dissolved in toluene and diluted to 0.2 mg/mL with toluene/methanol mixture (1:3, v/v). 15 μL ammonia solution (25%, HPLC grade) was then added into the solution to facilitate the deprotonation of the acid species and neutral nitrogen compounds to yield [M-H] ions. The analysis was performed on an Apex-ultra FT-ICR mass spectrometer (Bruker Daltonics, USA) equipped with an actively shielded 9.4-T magnet. Ions were generated with a negative-ion electrospray equipped with a 50-μL infused silica ESI needle. The samples were infused at a flow rate of 250 μL/h. The operating software was XMASS version 6.0 (Bruker Daltonics, USA). Each spectrum was composed of 64 scans.

### Glass Micromodel Test

**FIGURE 1:**
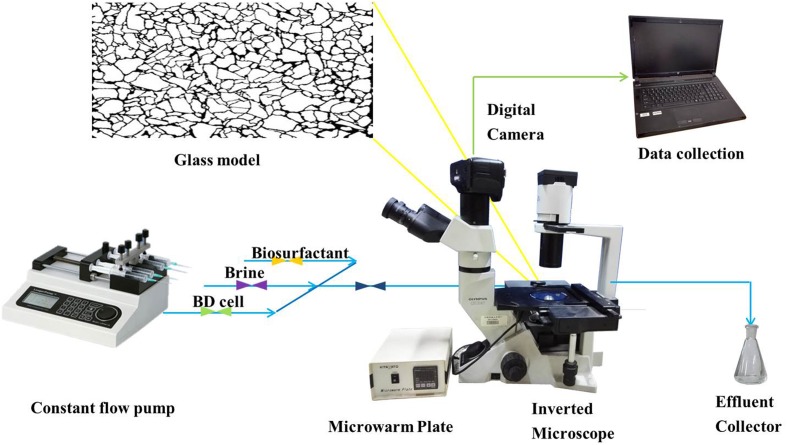
**Schematic of the glass micromodel experimental set-up**.

The setup of the micromodel is illustrated in **Figure 1**. The micromodel mainly consisted of a glass micromodel, a fluid injection system, a temperature controlling system, an optical and photography system and a monitoring computer. In this study, two glass micromodels (i.e., models A and B) were used in simulating oil recovery under reservoir conditions to examine the efficiency of strain BD in enhancing oil recovery. The manufacturing process of the micromodels has been described by Dunsmore et al. (2004). Briefly, a micromodel consisted of two glass plates, one with an interconnected, acid-etched network being annealed to the other, un-etched plate. The inner diameter of the core channels was approximately 20 μm (**Supplementary Figure S1**).

Micromodels were first saturated with crude oil at 90°C for 7 days. Then, fresh water was injected into the micromodels for 30 min to simulate the first water flooding. The flow rate of the flooding water was controlled at 8 μL/min with a LongerPump TS-1B constant flow pump (LongerPump, China). The pump and micromodels were connected with Masterflex 06409-13 tygon tubing (Saint-Gobain, France). The temperature of the flow system and micromodel was maintained at 35°C via a micro warm plate (Kitazato, Japan). Subsequently, cell-free fermentation broth containing biosurfactants was injected into model A for 10 min, followed by 20 min second water flooding. Into model B, bacterial colonies cultivated in MSM were continuously injected until no further oil was produced. Model B was then sealed for 36 h, followed by the second water flooding for 20 min. Photographs were taken during the flooding period every 2 min with an EOS550D digital single lens reflex camera (Canon, Inc., Japan), and the oil recovery was determined based on the change of pixel numbers of crude oil areas as evaluated using Photoshop CS3 (Xiao et al., 2013):

$$\text{Oil recovery = }\frac{{\text{Area}}_{\text{initial}}\,-{\text{Area}}_{\text{flooding}}}{{\text{Area}}_{\text{initial}}}\,$$

## Results and Discussion

### Screening of Biosurfactant Producer

The most promising biosurfactant producer, strain BD, was selected from 77 strains isolated from oil–water mixture samples because of its high performance in surface tension reduction and oil emulsification assays. The emulsification index E_24_ and the surface tension of the BD culture were determined to be 57% and 30.27 mN/m, respectively. The isolated BD strain is characterized as motile, gram negative, and MR negative. Moreover, strain BD can grow well in the temperature range of 20–55 °C, pH range of 4–10, and salinity range of 0–20%. The nearly complete 16S rRNA gene sequence (1382 bp) of strain BD was obtained, and the phylogenetic analysis showed that it has the highest 16S rRNA sequence similarity of 99.38% with *Acinetobacter junii* (Genbank accession number KT763370) (**Supplementary Figure S2**). The biosurfactant producer has been isolated from a wide diversity of environments including soil, sea water, marine sediments, oil fields, and even extreme environments (Yakimov et al., 1998). The biosurfactant producer isolated from reservoirs adapted better than others to the oil reservoir environment. Until now, numerous indigenous species have been known as biosurfactant producers and have been well-studied. Bacterial genera isolated from reservoirs, including *Pseudomonas. Bacillus. Enterobacter. Rhodococcus. Paenibacillus. Dietzia. Acinetobacter*, and *Brevibacterium*, were found to produce many kinds of surfactants (Najafi et al., 2011; Zheng et al., 2012; Khajepour et al., 2014). Some of these genera were found to produce different types of surfactants and have multiple species. For example, *Pseudomonas* was found to produce rhamnolipids, and in several cases produced lipopeptides (Xia et al., 2011, 2014). It is well-documented that *Acinetobacter* species generally produce exocellular polymeric bioemulsifiers. The well-known bioemulsans, for example, is a kind of a bioemulsifier produced by different species of *Acinetobacter*, such as *Acinetobacter calcoaceticus* RAG-1, *Acinetobacter radioresistens* KA53, *Acinetobacter junii* BB1A, *Acinetobacter calcoaceticus* BD4, etc. (Navon-Venezia et al., 1995; Toren et al., 2001; Sen et al., 2014). These microorganisms have shown to be very efficient in bioemulsifier producing. However, few researches are there about *Acinetobacter* isolated from reservoirs as a biosurfactant (small-molecule compound) producing microbe, although it is a dominant species in many reservoirs.

### Effects of Carbon and Nitrogen Sources on Biosurfactant Production

**FIGURE 2:**
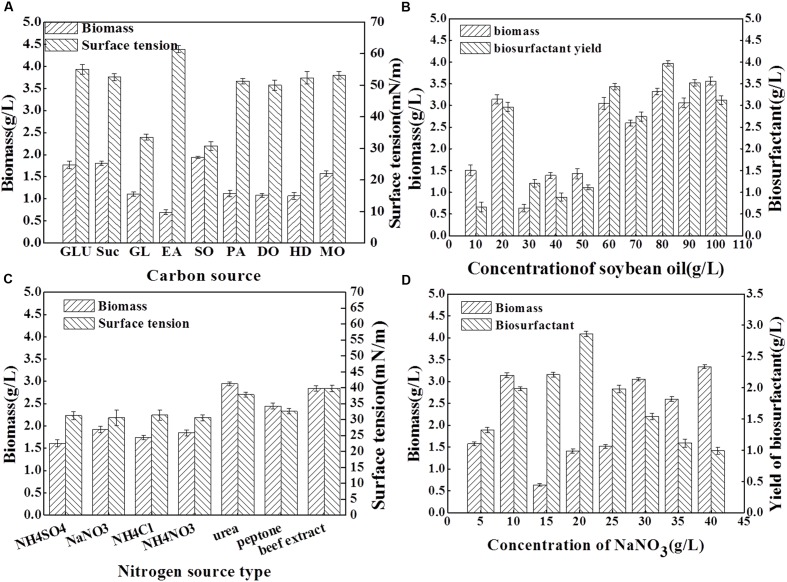
**Surface tension (mN/m), biomass yield (g dry weight L^-1^) and biosurfactant yield (g/L) obtained for the BD strain after 96 h cultivation in mineral salt solution with different carbon and nitrogen sources.** Results represent the average of three independent experiment ± standard deviation. **(A)** shows surface tension and biomass yield obtained for the BD strain cultivation in mineral salt solution with different kinds of carbon source. In **(A)**, GLU, glucose; Suc, sucrose; GL, glycerol; EA, ethanol; SO, soybean oil; PA, paraffin; DO, diesel oil; HD, hexadecane; and MO, molasses. **(B)** shows biomass yield and biosurfactant yield obtained for BD strain cultivation in mineral salt solution with different concentration of soybean oil as carbon sources. **(C)** shows surface tension and biomass yield obtained for the BD strain cultivation in mineral salt solution with different kinds of nitrogen source. **(D)** shows biomass yield and biosurfactant yield obtained for BD strain cultivation in mineral salt solution with different concentration of NaNO_3_ as nitrogen sources.

To determine the optimal medium that yields the highest biomass and biosurfactant production by *Acinetobacter junii* BD, the effects of various carbon and nitrogen sources were examined. The results are presented in **Figure 2**. As shown in **Figure 2A**, using the same concentration of carbon sources, and keeping other conditions constant, soybean oil as the sole carbon source resulted in the highest biomass yield; the surface tension of the medium was reduced from 70.22 to 30.75 mN/m which is the most significant among all of the carbon sources. Although, a high biomass yield was obtained when glucose and sucrose were used as sole carbon sources, the surface tension reduction of the medium was poor. Previous studies also reported that hydrocarbons and carbohydrates are the best carbon sources for biosurfactant production in other isolates (Gudiña et al., 2015). Many *Acinetobacter* isolates are regarded as bioemulsifier producers; Su et al. (2009) considered C_2_H_5_OH as the best carbon source for bioemulsifier growth and production. In the current study, soybean oil was determined to be the appropriate carbon source for the biosurfactant production with *Acinetobacter junii* BD. **Figure 2B** shows that higher concentrations of the carbon source enhanced biomass yield. Moreover, the maxima were observed when 10% soybean oil was added. However, the biosurfactant yield attained its highest value when 8% soybean oil was added, with a concomitant high biomass yield.

Comparing the effect of nitrogen source (8 g/L) on the biomass yield and surface tension (**Figure 2C**), the maximum biomass production was obtained when the strain was cultivated on urea. The maximum emulsification capacity and lowest surface tension were observed in the culture with NaNO_3_ as nitrogen source. Based on these results, it can be suggested that the optimum nitrogen source for BD growth differ from that for biosurfactant production. The presence of NaNO_3_ as the sole source of nitrogen with a concentration of 1.0% (w/v) in the soybean oil-containing MSM resulted in a high level of biomass and biosurfactant production (**Figure 2D**).

### Thin-Layer Chromatography (TLC) Analysis of the Purified Biosurfactants

**FIGURE 3:**
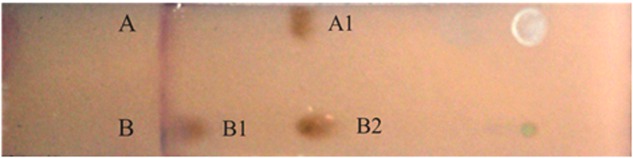
**The thin-layer chromatogram of crude biosurfactant.** Lanes **(A,B)** were developed with phenol-sulfuric acid method to detect glycolipid. **(A)** The thin-layer chromatogram of rhamnose; **(B)** the thin-layer chromatogram of biosurfactant produced by BD when utilizing soybean oil as carbon source. Brown color is positive.

Considering the significant reduction of surface tension in the current study, it seems that the strain BD synthesized low-molecular-weight biosurfactants in the MSM with soybean oil as the sole carbon source. As previously described, low-molecular-weight biosurfactants are generally glycolipids, phospholipids and lipopeptides (Abdel-Mawgoud et al., 2010; Gudiña et al., 2013). Two types of biosurfactants exhibited positive reactions with two brown reagents of a phenol sulfate solution (**Figure 3**). This observation indicated that different homologous series might belong to glycolipids that consisted of sugar and lipid moieties (Juliana et al., 2013).

### Diversity of the BD Biosurfactants

**FIGURE 4:**
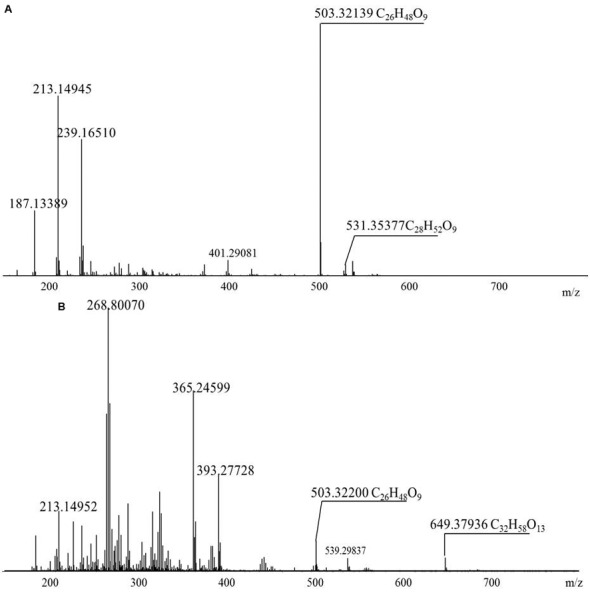
**Negative-ion electrospray FT-ICR mass spectra of the purified biosurfactant.** **(A)** Biosurfactant eluted with 1:1 methanol:chloroform; **(B)** 1:2 methanol:chloroform.

The diversity of the biosurfactants produced by *Acinetobacter junii* BD is illustrated in **Table 1**. A series of singly charged negative ions were observed in the BD biosurfactant mix (**Figure 4A**). The mass under the negative mode exhibited deprotonated molecular ions at m/z 503.32139, which was considered to be the most abundant ion in the TLC fraction of the biosurfactant eluted with 1:1 methanol: chloroform. This ion peak was analyzed with the FT-ICR-MS data analysis software and was shown to correspond to C_26_H_48_O_9_, which generated three hits in the ChemSpider database. By reviewing the TLC analysis and searching against the ChemSpider database, it was determined that the C_26_H_48_O_9_ molecule was 3-({3-[(6-deoxy-α-L-mannopyranosyl) oxy]decanoyl}oxy)decanoic acid, a type of mono-rhamno-di-lipidic rhamnolipid (Abdel-Mawgoud et al., 2010). Similarly, the ion at m/z 531.35377 was assigned to C_28_H_52_O_9_, which may belong to homologs of this mono-rhamno-di-lipidic rhamnolipid.

**Table 1:** The diversity of biosurfactants produced from strain BD.

m/z	Formula	DBE	Metabolite class
503.32139	C_26_H_48_O_9_	3	Mono-rhamno-di-lipidic congeners
531.35377	C_28_H_52_O_9_	3	Mono-rhamno-di-lipidic congeners
557.36973	C_30_H_54_O_9_	4	Mono-rhamno-di-lipidic congeners
529.33773	C_28_H_50_O_9_	4	Mono-rhamno-di-lipidic congeners
475.2911	C_24_H_44_O_9_	3	Mono-rhamno-di-lipidic congeners
473.27579	C_24_H_42_O_9_	4	Mono-rhamno-di-lipidic congeners
649.37936	C_32_H_58_O_13_	4	Di-rhamno-di-lipidic congeners
677.41079	C_34_H_62_O_13_	4	Di-rhamno-di-lipidic congeners
621.34781	C_30_H_54_O_13_	4	Di-rhamno-di-lipidic congeners
419.22967	C_20_H_36_O_9_	3	Mono-rhamno-di-lipidic congeners
479.24932	C_22_H_40_O_11_	3	Di-rhamno-mono-lipidic congeners


In the TLC fraction of the biosurfactant sample eluted with 1:2 methanol: chloroform (**Figure 4B**), the molecular ions at m/z 649.37936 corresponding to C_32_H_58_O_13_ garnered three hits in the ChemSpider database. Based on TLC analysis and a search against the ChemSpider database, C_32_H_58_O_13_ was assigned to 3-[(3-{[6-deoxy-2-*O*-(6-deoxyhexopyranosyl) hexopyranosyl] oxy} decanoyl) oxy] decanoic acid, a type of di-rhamno-di-lipidic rhamnolipid. C_32_H_58_O_13_ was the most abundant di-rhamnolipid in the BD biosurfactant mix. Molecular ions at m/z 503.32139 were also detected. This result implied that C_26_H_48_O_9_ was the main biosurfactant produced by BD. The unique ion at m/z 531.35355 was assigned to C_28_H_52_O_9_, which was a homolog of the mono-rhamno-di-lipidic rhamnolipids.

*Acinetobacter* strains produce groups of compounds including high-molecular-weight bioemulsifiers, such as emulsan, alasan, and exopoly saccharide, which are effective in stabilizing oil-in-water emulsions (Navon-Venezia et al., 1995; Toren et al., 2001; Sen et al., 2014). The *Acinetobacter* strain in this study exhibited high levels of biosurfactant production. However, the molecular mass of the purified biosurfactants indicated the presence of more than 10 major and minor congeners of rhamnolipids. The largest portion of the biosurfactants was composed by the mono-rhamnolipids. Microorganisms can produce rhamnolipid homologs with various fatty acid chain length and number of rhamnose units. According to Nitschke et al. (2005), rhamnolipids with a longer fatty acid chain possess stronger hydrophobicity.

### Analysis of Polar Components during the Biodegradation of Crude Oil

The mass spectra of the negative ions obtained from the ESI FT-ICR-MS analysis of the crude oil showed that the contour of the molecular weight spectrum of the residual crude oil shifted toward lower molecular weights during degradation by strain BD. This finding indicates that biodegradation converts larger molecules into smaller ones (**Supplementary Figure S3**).

#### Compound Class Distribution

**FIGURE 5:**
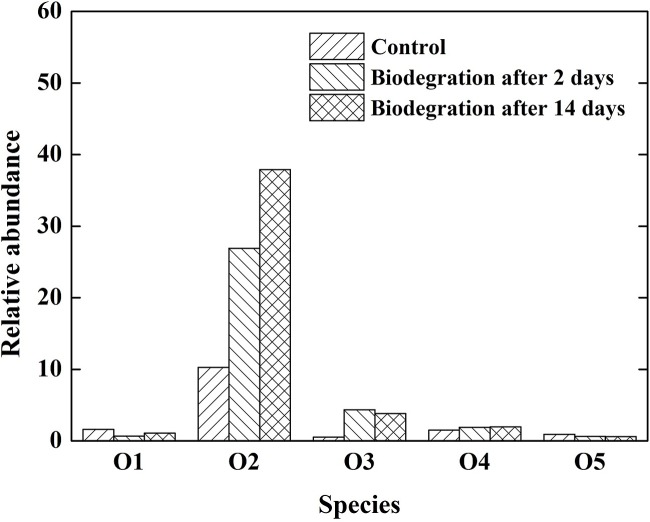
**Relative abundance of different heteroatom classes (number of heteroatoms) derived from negative-ion ESI FT-ICR mass spectrum of the crude oil**.

The importance of oxygenated polar compounds as interfacial active components in crude oil is widely acknowledged as one of the primary causes of emulsion stability in crude oil mixtures (Acevedo et al., 1999; Pauchard et al., 2008). Oxygenated polar compounds also play an important role in wettability alteration. The distribution of heteroatom-containing species in the crude oil subject to various biodegradation is presented in **Figure 5**. The O2 class was predominant, followed by O1, O5, and O3 heteroatom-containing classes. Similarly, the O2 class was the most abundant species in the crude oil after biodegradation, then the abundance decreased in the sequence of O3, O4, O1, and O5. The abundance of O2 species increased with increasing the extent of biodegradation, which is consistent with previous report (Kim et al., 2005). In this study, the increase in O2 species was the most predominant, and its abundance increased from 12.3 to 33.2% after 14 days of biodegradation. The O2 species in the crude oil were mainly carboxylic and naphthenic acids. An important mechanism by which emulsions are stabilized in crude oil systems is through the adsorption of carboxylic acids and their anions. The acids in crude oil can be adsorbed to the oil–water interface and dramatically lower the IFT (Hoeiland et al., 2001).

#### Distribution of *O*-Containing Compounds

**FIGURE 6:**
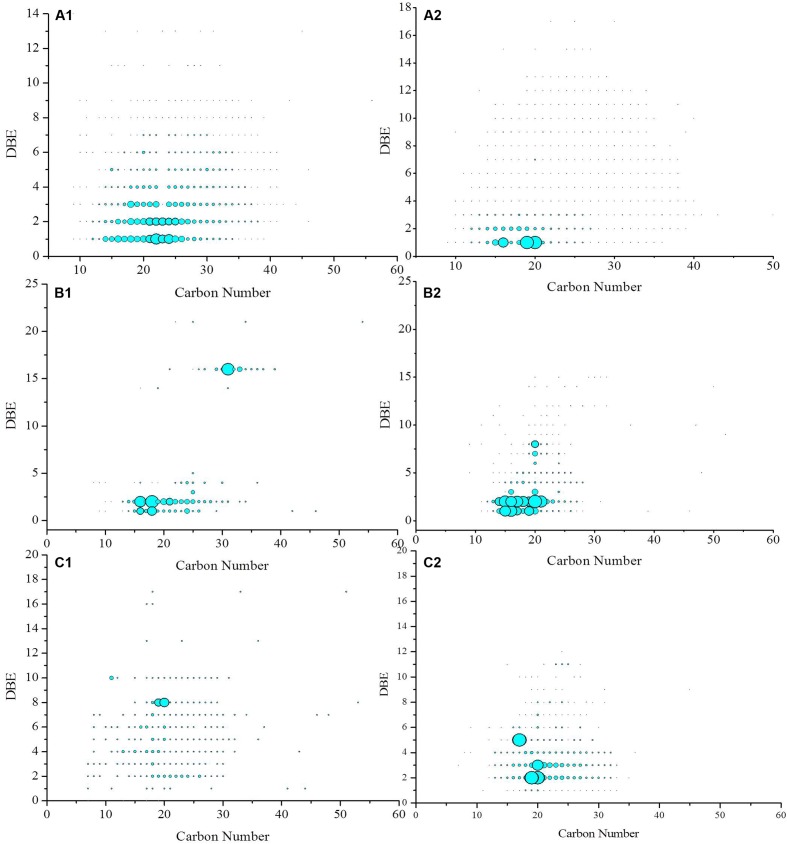
**Iso-abundance plots of DBE versus carbon number of O2 **(A1,A2)**, O3 **(B1,B2)**, and O4 **(C1,C2)** class species in crude oil before **(A1,B1,C1)** and after biodegradation **(A2,B2,C2)**.** The size of circles corresponds to the relative abundance of O2, O3, and O4 species in the spectra.

The iso-abundance plots of double bonds equivalent (DBE) versus the carbon number of the O2, O3, O4 class species in crude oil before and after biodegradation are shown in **Figure 6**. The distribution of the O2 species with DBE of and carbon number provides information on the effects of biodegradation on oil displacement. The O2 species with DBE of 1 are fatty acids, whereas those with DBE of 2–7 correspond to 1- to 6-ringed naphthenic acids (Pan et al., 2013). In contrast, O2 species with a high DBE value and low carbon number are likely to be multi-ring naphthenic acids, aromatic acids, or phenols with multiple hydroxyl groups (Shi et al., 2010b). In this study, before biodegradation, the DBE of the O2 species in the crude oil was primarily in the range from 1 to 4, and their carbon numbers was mainly in the range of 10–30. However, after biodegradation by strain BD, the DBE of the O2 species narrowed to 1 and 2, and their carbon number mainly ranged from 14 to 22. It has long been known that acids in crude oil can adsorb to a crude oil–water interface and dramatically lower the IFT (McCaffery, 1976). Emulsion is stabilized by naphthenates through the formation of alkaline earth soaps, particularly calcium naphthenate, by polyvalent acids (Baugh et al., 2004; Lutnaes et al., 2006).

The relative abundance of O3 species increased during biodegradation. The relative abundance of O3 species with DBE of 1 and 2 significantly increased. These constituents are likely hydroxyl fatty acids. The O4 species in the crude oil before biodegradation was sparsely distributed in the DBE range of 1–18 and carbon number between 7 and 53. After biodegradation, the species was in the DBE range from 1 to 12 and carbon numbers from 7 to 35. The O4 species with a DBE of 2 had the highest relative abundance and were likely saturated fatty dibasic acids. The species with a DBE of 5 also became the dominant compounds after biodegradation. These species were likely dicarboxylic acids with three naphthenic rings (Mapolelo et al., 2011).

The acid components in crude oil are mainly alkyl carboxylic, alkyl benzene carboxylic, naphthenic, and fused aromatic ring acids. Some of these components may be large molecular species with a combination of fused aromatic rings, alkyl side chains, and other chemical moieties with carboxylic acid pendant groups. The acid components in crude oil can be ionized at the oil–water interface to form the anion of the acid and to dramatically lower the IFT (Gao et al., 2010). This occurrence leads to the stabilization of interfaces and emulsion. During biodegradation, the content of fatty acids, naphthenic acids, multi-ring naphthenic acids, aromatic acids, and phenols with multiple hydroxyl groups rapidly increases. The microorganisms attached to the solid surface and those that can degrade crude oil on the solid-oil surface can sweep oil from the rock and improve the mobility of petroleum.

### Analysis of the Glass Micromodel Test

Glass micromodels were used to evaluate the effect of microbial enhancement of the oil recovery. **Table 2** compares the oil recovery with different injection methods in glass micromodel tests using bacteria and biosurfactants. The final oil recovery when only formation water flooding was applied was 54.6% after 60 min. A 2.8% enhancement of oil recovery was achieved by flooding with bacterial broth for 10 min. This value was further increased with a second period of formation water flooding after a 36 h shut-in period, resulting in a 9.6% improvement in total oil recovery. In another experiment, the oil recovery was increased by 13.4% after biosurfactant flooding. The result shows that strain BD and its metabolite have a great potential for enhancing oil recovery.

**Table 2:** Oil recovery of different injection methods in the micromodel.

Test project	Flooding rate	Oil displacement efficiency (%)		
		First water flooding	Second water flooding	MEOR
Model A with biosurfactant flooding	8 μL/min	49.7	63.1	13.4
Model B with bacteria flooding	8 μL/min	54.6	64.2	9.6


Biosurfactants have a great potential for enhancing oil recovery, which is also demonstrated in the literature. In the water flooding experiments investigating the MEOR potential of *Rhodococcus ruber* Z25, Zheng et al. (2012) achieved improvement of oil recovery by 8.9–25.8%. Application of biosurfactant producing strain *Enterobacter cloacae* increased the oil recovery up to 24.5% (Khajepour et al., 2014). Xia et al. (2011) reported 14.3% enhancement compared with the control condition using the biosurfactants of *Pseudomonas aeruginosa* in their oil recovery experiment. The results of oil recovery by this study show that both the BD strain and its metabolites are effective in increasing oil recovery.

Rabiei et al. (2013) compared the results of oil recovery from *in situ* and *ex situ* tests and showed that in the quick-flooding tests, the incremental oil recovery in the *ex situ* process was higher than in the *in situ* process due to the partially purified biosurfactant that was used in the *ex situ* test. The results of this work are consistent with those of Rabiei et al. (2013). Moreover, by means of IFT and wettability measurements, Rabiei et al. (2013) concluded that IFT reduction was predominant in the quick-flooding test, whereas in the shut-in test, both IFT reduction and wettability contributed to improved oil recovery.

**FIGURE 7:**
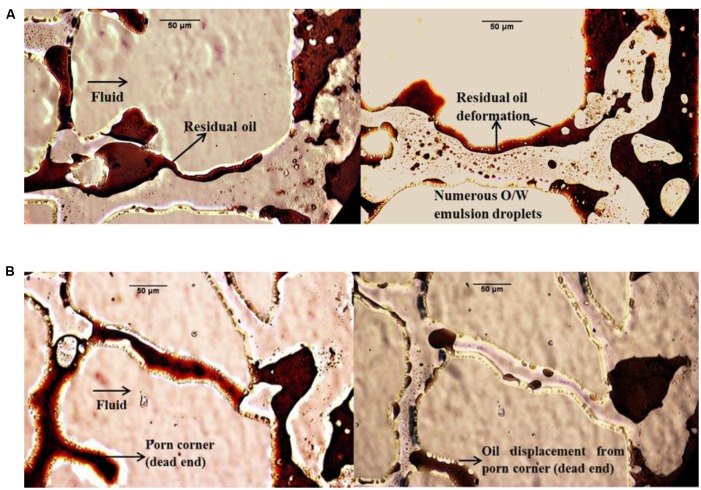
**Microphotograms showing the fluid distribution after treatment with supernatant (cell free) solution in micromodel **(A)** and bacterial solution in micromodel **(B)****.

In this work, many photos were gathered to directly assess the MEOR mechanisms in the micromodel system. **Figure 7** compares the fluid distribution in the glass micromodels after flooding with bacterial and biosurfactant solutions for different stages. The microphotogram on the left hand side of model A shows that after water flooding, the interface between water and the residual oil was rigid, which in turn made the residual oil difficult to move further. Oil emulsification and deformation were commonly observed during both bacterial and biosurfactant flooding. The biosurfactants produced by the bacteria decreased the IFT between petroleum and formation water. The lowered IFT resulted in emulsification and deformation of the oil confined in the pores, and the oil became significantly easier to transport. This means that IFT reduction and resultant oil emulsification was the main mechanism by which oil recovery was enhanced by bacterial and biosurfactant flooding.

Wettability reversal either from oil-wet to water-wet state or from water-wet to oil-wet state increases oil recovery (Mehranfar and Ghazanfari, 2014). Sarafzadeh et al. (2013) reported that bacterial adhesion and biofilm formation can convert wettability from the neutral- or oil-wet state to the water-wet state. After a proper shut-in time period, more tertiary oil was recovered due to the wettability alteration mechanism. In this study, wettability alteration was observed after the shut-in period in bacterial flooding (**Figure 7B**). Comparing the oil area within the same dead pore in the two microphotograms of model B, it can be seen that oil displacement took place to some extent. **Figure 7B** shows that microorganisms moved into the dead pore and swept the oil, which was almost immoveable during the secondary water flooding. The wettability was changed by the attachment of the bacteria to the solid surface and the adsorption of biosurfactants and polar compounds on the solid surface.

In general, strain BD and its biosurfactants enhanced the oil recovery process after the first water flooding and intensified the second water flooding in the glass micromodels. The micromodel test showed that IFT reduction, wettability alteration, and the mobility of microorganisms were the dominant mechanisms enhancing the oil recovery.

## Conclusion

The novel rhamnolipid-producing strain *Acinetobacter* sp. BD was isolated from the formation water of an oil reservoir. This strain showed significant rhamnolipid-producing and oil-degrading capability. The biosurfactants produced with the optimal carbon and nitrogen sources were identified through FT-ICR-MS as different isoforms and homologs of rhamnolipids. Strain BD and its biosurfactants have great potential to enhance oil recovery, as demonstrated by flooding tests with a glass micromodel.

## Author Contributions

Conceived and designed the experiments, wrote the manuscript: HD. Performed the experiments: HD, HhD, PZ, KL. Analyzed the data: HD, WX. Contributed reagents and materials: ZZ. Contributed analysis tools: SS, CL. Helped perform the analysis with constructive discussions: YS, GZ. Approved the final version: ZS.

## Conflict of Interest Statement

The authors declare that the research was conducted in the absence of any commercial or financial relationships that could be construed as a potential conflict of interest.
