# Supplementary material for: Rhamnolipids Produced by Indigenous Acinetobacter junii from Petroleum Reservoir and its Potential in Enhanced Oil Recovery
Source: Front Microbiol. 2016 Nov 7;7:1710. doi: 10.3389/fmicb.2016.01710 (PMC5097926; doi:10.3389/fmicb.2016.01710)
Supplement: Supplementary file 1 [file Presentation_1.PDF]

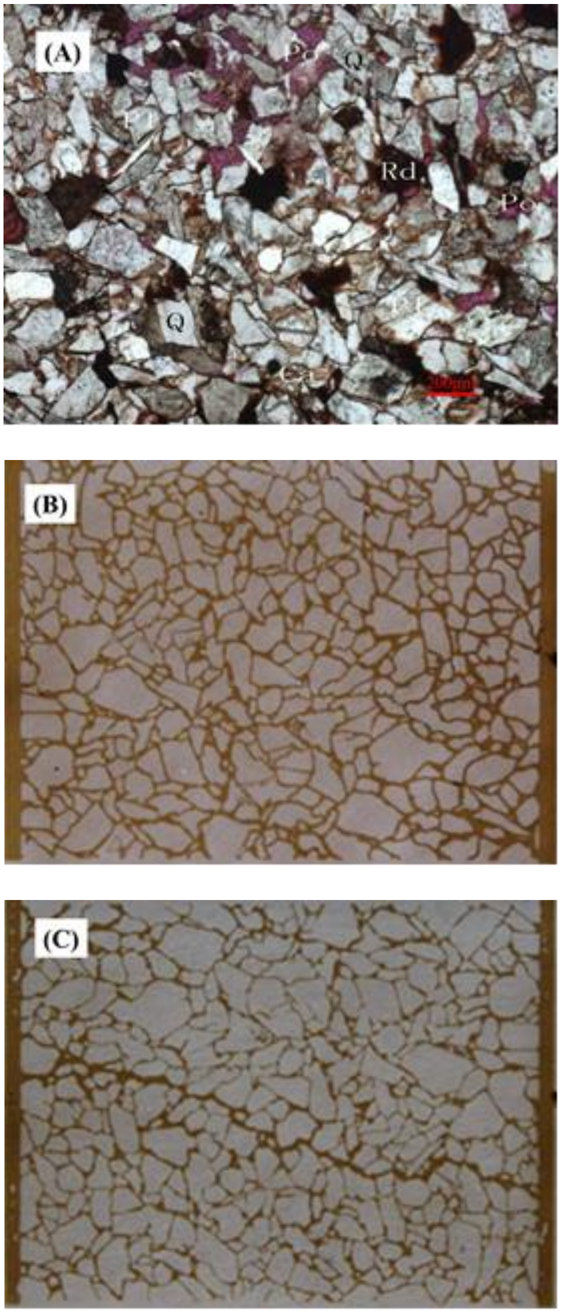

Supplementary Figure 1. Microphotographs of the rock core and micromodels with different patterns. The lengths of the micromodels were 5cm and the widths were 2.5cm. (A) Original low permeability reservoir core; (B) (C)Micromodel saturated by crude oil.

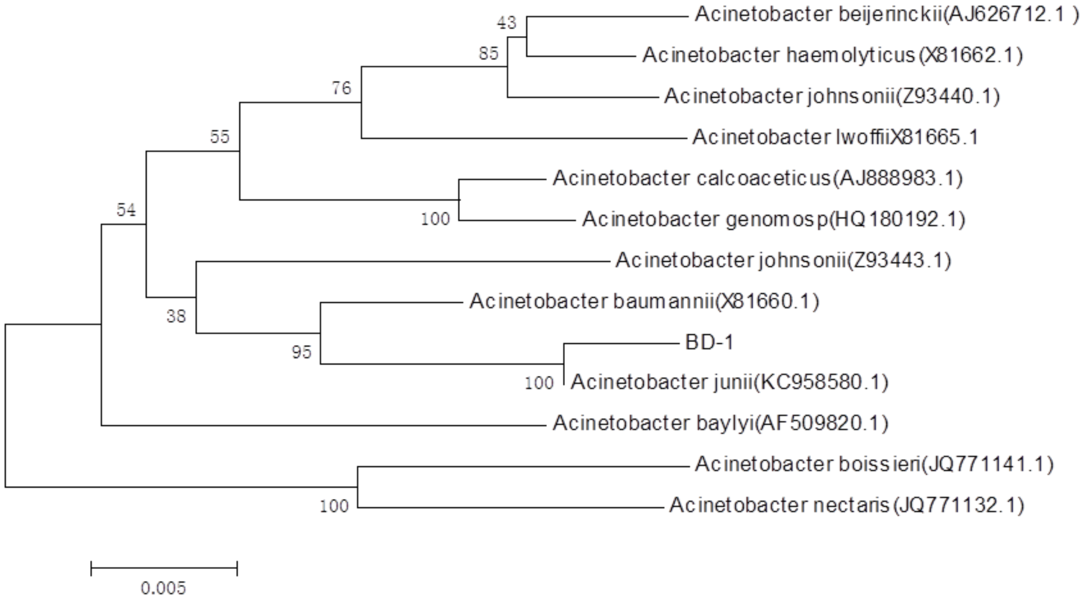

Supplementary Figure 2. Phylogram based on the comparison of 16S rRNA sequence of other bacteria which have closed relationship of 16S rRNA of biosurfactant-producing strain BD.

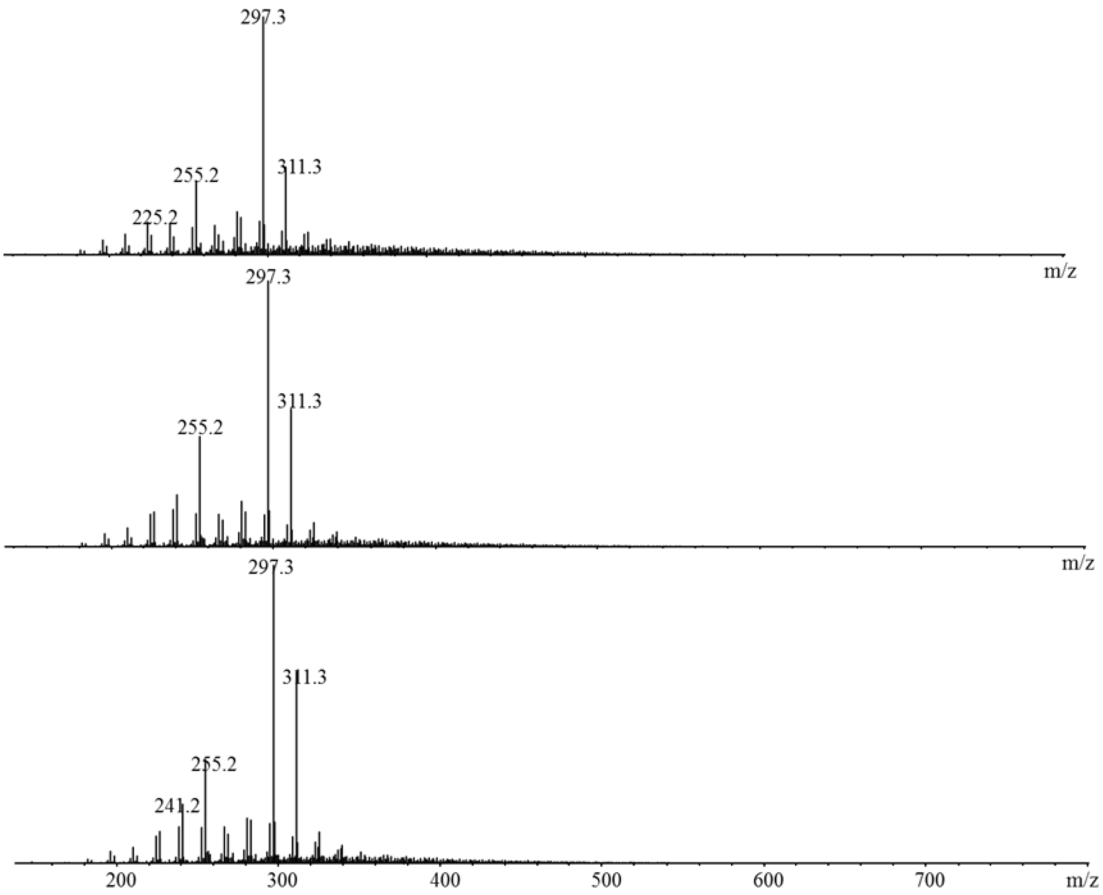

Supplementary Figure 3. Negaive-ion electrospray FT-ICR mass spectra of the crude oil during the biodegradation.
